# Renal Apoptosis in Male Rats Induced by Extensive Dietary Exposure to Ochratoxins

**DOI:** 10.3390/ijms26104553

**Published:** 2025-05-09

**Authors:** Peter Mantle, Rohit Upadhyay, Diana Herman, Vecihi Batuman

**Affiliations:** 1 Centre for Environmental Policy, Imperial College London, London SW7 2AY, UK; 2 Section of Nephrology and Hypertension, John W. Deming Department of Medicine, Tulane University School of Medicine, New Orleans, LA 70112, USA; rupadhyay@tulane.edu (R.U.); vbatuman@tulane.edu (V.B.); 3 Pathology Department, County Hospital, 300736 Timisoara, Romania; diaherman@yahoo.com

**Keywords:** ochratoxins, dietary exposure, renal cancer, maximum lifetime exposure, urothelial carcinogenic factor, aristolochic targets

## Abstract

During the 70 years since the Balkan endemic nephropathy was recognised, failure to make a universal diagnostic cause continues for some critical researchers. Claims for cause by toxic molecules from microorganisms and/or plants are difficult to verify experimentally in retrospect, partly because no lifetime human experimentation is possible and partly since no experimental source is a convincing model. Apoptosis as a primary step in human nuclear decline has been a source of experiments for many years. Now, one has been used, which employs the detection of abnormal nuclear hydroxyls by using an Abcam histology protocol. Recent access to a few human tissues diagnosed for the Balkan nephropathy has enabled preliminary exploration to publish some positive human manifestations of apoptosis. Parallel use and positive findings have also now illustrated applications to experimental rats’ kidneys after daily dietary exposure to ochratoxin A in lifetime experiments. Focus on renal cortical nephrons and their nuclei reveals a TUNEL-stained pattern with intensity linked to many months of the sub-clinical ochratoxin dietary exposure. The pattern survives long after exposure, but where experimental rats have internally developed cancer did not arise outside the kidney. Our experimental rat findings could not attribute the mechanism for the very different human urothelial tumours to traces of dietary ochratoxin A, but the present study encourages the exploration of more archived Balkan nephropathy renal cases to predict the focal urothelial origin of early tumours.

## 1. Introduction

The Balkan endemic nephropathy (BEN), which became recognised about seventy years ago first in Bulgaria, has since challenged the explanation for which plant and/or microbial metabolites might be recognised as the cause. The topic has since stimulated extensive research publication. Even indirectly in the 1990s, the chronic illness of some women in Belgium occurred during their accidental renal poisoning from a medication for their over-weight control [1]. Accidental incorporation of the plant metabolite aristolochic acid in the Belgian informal overweight-medication component allowed some indirect conclusions that an acute example of a natural mechanism, somewhat-like BEN, was being illustrated accidentally. Unfortunately, the subsequent accurate quantitative analysis of aristolochic acid in that medication proved difficult, both in the London School of Pharmacy and in the UK Royal Botanic Garden in Kew. Nevertheless, Belgian experimental treatment then of female rabbits was by frequent intraperitoneal injection of aristolochic acid for up to 3 years [2]. It failed clearly to explain the cause of BEN.

Meanwhile, discovery of the fungal metabolite ochratoxin A (OTA) in South Africa in the 1960s [3] became soon recognised as the cause of seasonal porcine nephropathy, which had frequently affected the Danish bacon industry [4]. A major pharmacological study of OTA in the USA in the 1980s defined its causation of male Fischer rat renal tumours from adult lifetime oral dosing [5]. Alerting attention to revealing a possible risk of human kidney cancer then came from within the International Agency for Research on Cancer [6], showing experimentation describing a possible risk factor in the BEN context. Further adult lifetime gavage-dosing of Dark Agouti and Lewis rats of both genders with OTA then occurred in Hannover, Germany, in the 1990s. It provided kidneys for analysis of DNA adducts which were found associated with renal tumours, mainly in males [7]. Focus on these findings subsequently confirmed their predictive value concerning potential human risk from the OTA contamination of agricultural produce, foodstuffs and beverages. Although unconfirmed, DNA adduction might produce human health risks from only slight natural OTA contamination of some foodstuffs during a protracted lifetime. The subsequent publication of experimental evidence supporting any uncertainty of significant adduct formation in response to OTA intake is at least open for the need of confirmation, according to recent European Food Safety Authority recommendations [8].

More recently [9], the description of a small DNA structural change in kidneys of BEN cases has been explored in renal tumours associated with some BEN cases. However, it has hardly explained that such tumours seemed only of urothelial occurrence, and the start of the general silent renal decline was not explained, leading eventually to BEN diagnosis in the quite-often absence of any urothelial tumour.

Apoptosis is a topic that can be explored to consider the gentle cause of BEN as a human disease in certain rural villages. The design for its special histological exploratory technique has been developed in Cambridge, UK, by Abcam. Partly, it can focus on TUNEL detection to focus on very early stages in nuclear decline and has recently been explored in this context [10]. It involved applying the exploration of TUNEL changes to natural late-stage BEN tissues from some Serbian kidneys surgically removed because of their urothelial tumour. Somewhat similarly, experimental lifetime rat renal tissues given dietary OTA were also available in London. Continued collaboration to explore TUNEL changes has yielded the present description.

## 2. Results

### 2.1. Carcinogenic Dose of Ochratoxin for Life for Three Fischer Rats

The description of histological renal evidence of the long-term daily dietary exposure to ochratoxin A is readily available elsewhere for the illustrated male rats [11]. Three Fischer rat examples were selected here to explore the daily intake of 300 microgram ochratoxin A/kg in body weight and to illustrate specific features of renal cortex and associated renal tumour in response. General rat renal tumour illustration is also available [12] for adult life-time exposure to OTA which assures that first year exposure is sufficient to cause renal tumour eventually.

**Figure 1:**
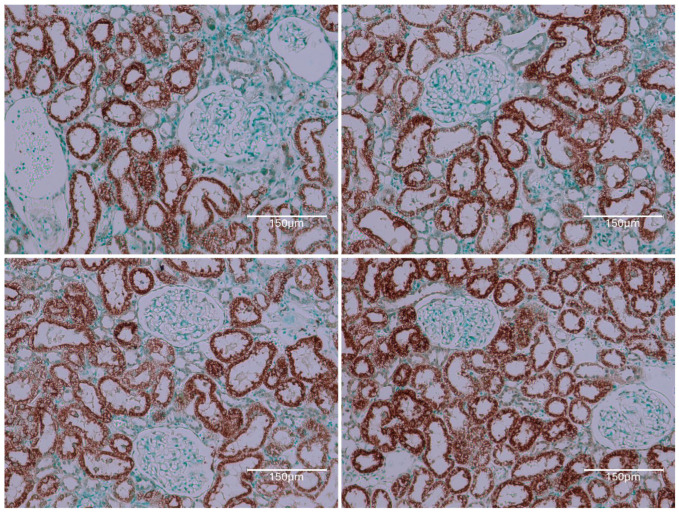
Three representative cortical examples of renal histological contrasts are extensive and attributed to this first year’s ten months of dietary OTA treatment for this rat 1. Contrast is illustrated by this extensive TUNEL staining of renal cortical proximal convoluted nephrons but apparently without retained focus on its nuclei through the second year of life. This is a critical illustration. No tumour is evident in these examples across this stained illustration, but, in any case, the eventual tumour is not expected to have yet originated in this tissue section at the 10-month treatment expiry stage. Renal carcinogenesis is probably already prescribed to occur eventually but not necessarily within this cortical region. Bars 150 μm.

Thus, for the present context, the renal illustration in Figure 1 can be regarded as at least the product of the first half of a lifetime’s continuous exposure to OTA. It also actually illustrates what then survived about half a year’s subsequent decline of circulating OTA. During the years of OTA experimentation at Imperial College, there never was any plan to shorten about 10 months of first year exposure to OTA to guarantee the initiation of renal cancer. It could have been useful if the present revelation of a complex pattern of renal tissue responses to continuous circulating OTA had been explored experimentally over a whole lifetime. That may of course now be recommended. However, in viewing the experimental findings here, it can be assumed that differentiation of the dynamics within cortical nephrons’ TUNEL staining had been a gradual process, now illustrated for rat 1 in Figure 1.

**Figure 2:**
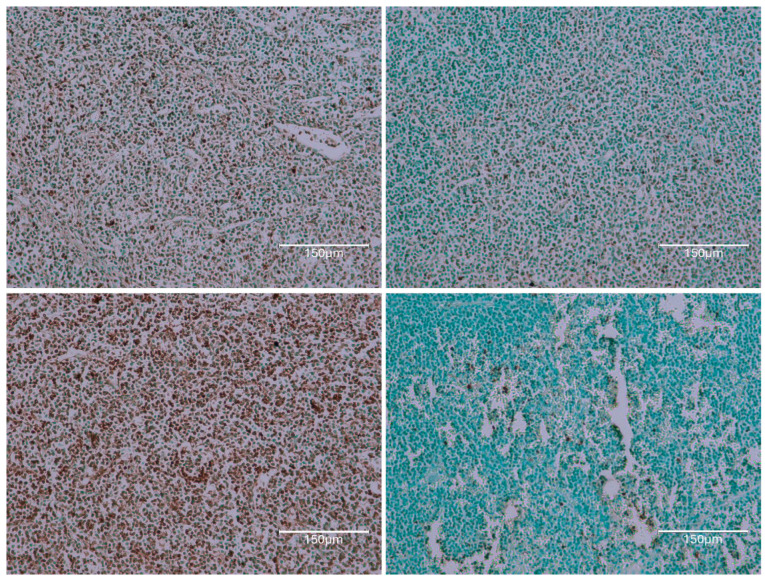
OTA for life (nearly two years). No obvious kidney in the section, but TUNEL staining is scattered possibly on nuclei in apparently older dense renal-tumour tissue. Intense light-green stained tissue is presumed to be of relatively younger tumour but already has nuclei readily stained as TUNEL-affected. Bars 150 μm.

Rat 2 received the same, 10-month, OTA diet in its young adult life. Illustration in Figure 2 focuses on examples of tumour tissue reflecting the evidence of nuclear staining, revealed after application of the Abcam TUNEL detection kit (ab206386; Abcam, Cam-bridge, MA, USA). Its pattern of cortical histopathology was, as expected, very similar to that illustrated in Figure 1.

The third rat example illustrates kidney tissue near the tumour of Figure 2 in the same rat, providing proof of complementary OTA changes throughout the adult lifetime exposure of renal cortex nephrons to OTA. The notable evidence here is the staining of nuclei linked with continued renal exposure to the lifetime circulation of OTA, contrasting with all pictures in Figure 1 and emphasising consistent pathology response. Contrasting different histology revealed for the cortical kidney component of this rat, by comparison with rat 1 above, is emphasised by the frequent TUNEL-stained nuclei persisting through the second year of life, while a renal tumour was arising elsewhere within the kidney.

These three high-dose rats, with at least the first year’s exposure to dietary OTA, combine to demonstrate a marked persistent staining response in cortical nephron cytoplasm, containing specific nuclear staining resembling apoptosis. Nuclear evidence de-clined or disappeared by the end of life in the cases where OTA was given only in the first year. This tumour example showed extensive apoptotic staining characteristic of positive responses to the Abcam 206,386 methodology. All samples were randomly selected from various regional sources to explore the relevance of mid- and long-term exposure to OTA in the current study.

### 2.2. Low-Dose Ochratoxin for Life for Three Fischer Rats

Rats were given dietary OTA daily throughout adult life at 50 microgram/kg of body weight [13], sustained on a much lower OTA diet than illustrated in the rats above. They naturally responded eventually with less intense TUNEL staining although still focused in the renal cortex and in tumour. Response to the modest OTA exposure, only 12% of the published 34 rats, eventually gave renal carcinoma.

**Figure 3:**
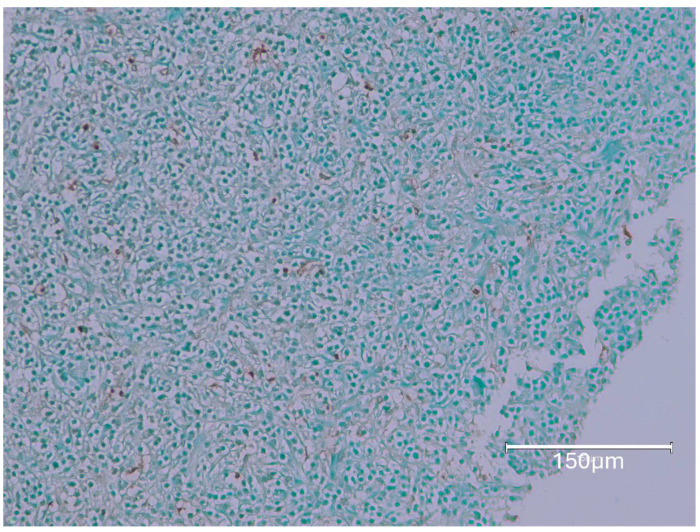
Rat 1A. Renal tumour with widespread evidence of TUNEL staining attributed to some of its nuclei. Bar 150 μm.

**Figure 4:**
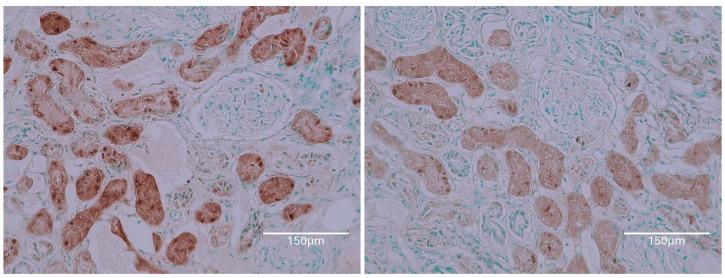
Extensive OTA for adult life (nearly two years). Kidney section in oblique aspect with oblique nephrons TUNEL-stained lightly brown but with black nuclei (TUNEL + ve, like apoptosis). Contrasting different histologies revealed for the cortical kidney component of this rat, by comparison with rat 1 above, is emphasised by the frequent TUNEL-stained nuclei persisting through the second year of life, while a renal tumour was arising elsewhere within the kidney. Bar 150 μm.

**Figure 5:**
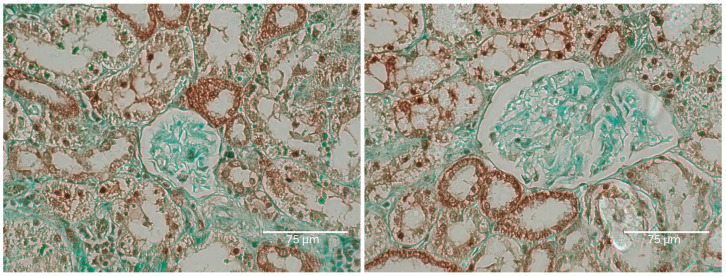
Rat 1B. Two renal cortex examples of particularly prominent TUNEL staining of descending nephron nuclei within some less-stained nephron epithelium. Slight nuclear-like TUNEL stains are even in Bowman’s capsules and their contents; this might only reflect the natural near demise of the rat. Bars 75 μm. Rat 2 in this section, also given the six-fold lower OTA diet, survives for nearly 2 years and still responds with a relatively faint renal histopathology.

**Figure 6:**
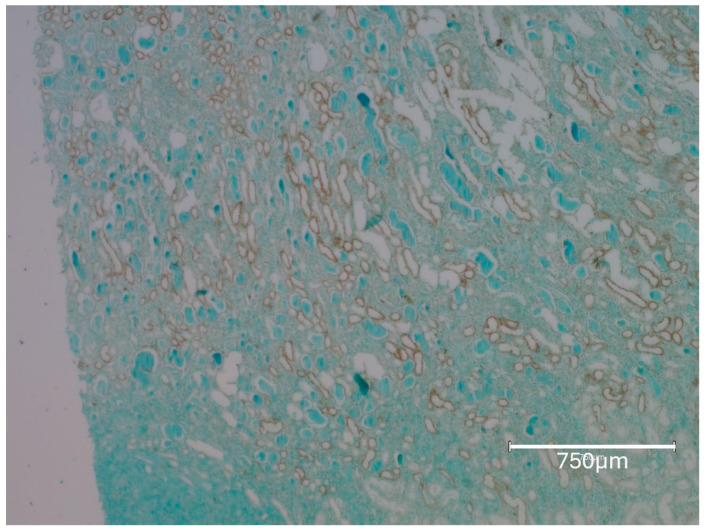
Rat 2A. Nephron histopathology staining is easily illustrated by TUNEL staining in a region across kidney cortex. The small edge of renal adenoma in the lower left corner here remains unstained for TUNEL; this emphasises staining contrast for the present study, failing to stain renal tumour. Bar 750 μm.

The first rat in this group, exposed to the six-fold lower OTA intake for 76 weeks before demise, eventually revealed a small, renal tumour with occasional, black-stained apoptotic nuclei (Figure 3, rat 1A). Illustration of the same comprehensive TUNEL staining as expressed in Figure 1, Figure 2 and Figure 4, focusing there on their six-fold greater daily OTA dose, confirms the specific anatomic foci for again recognising renal apoptosis detection by the Abcam histology kit in these experimental-rat tissues. Detailed display also of the TUNEL staining in the renal cortex after the 76 weeks of this lower OTA consumption is illustrated later in Figure 5 where background tissue seems generally to have avoided retaining TUNEL staining. Nephron histopathology was clearly demonstrated through TUNEL staining in a region of the kidney cortex (Figure 6). Notably, the small edge of a renal adenoma in the lower left corner remained unstained by TUNEL, high-lighting the contrast between the cortical nephron cells and the renal tumor. This lack of staining in the renal tumor emphasizes the specificity of TUNEL staining in detecting apoptotic cells, which does not extend to tumor tissue in the present study.

**Figure 7:**
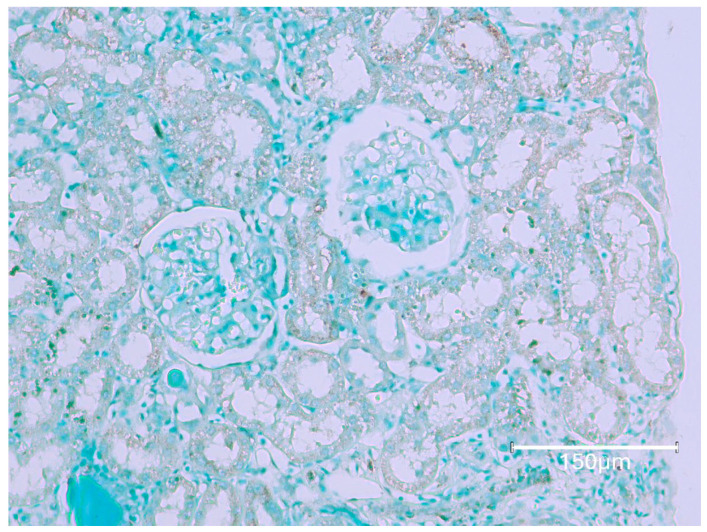
Rat 2B. Also, mild cortical response to 100 weeks of mild OTA consumption. Renal cortex illustrations with slight nephron staining, evident from magnification. Bar 150 μm.

Figure 7 illustrates the mild cortical response observed in Rat 2B after 100 weeks of moderate OTA consumption. The renal cortex shows slight nephron staining, which be-comes more apparent under magnification, indicating subtle changes in nephron structure.

**Figure 8:**
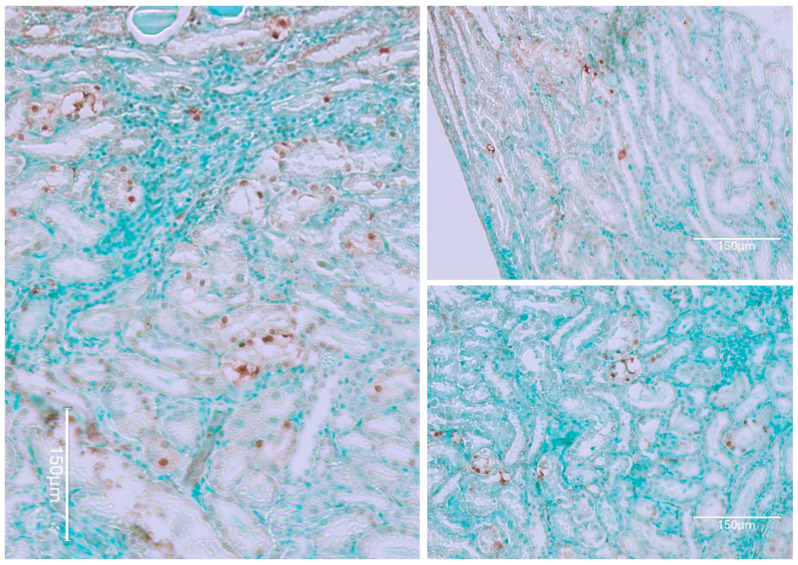
Rat 3. OTA exposure for 85 weeks. Occasional clusters of TUNEL-stained nuclei in a non-cortical region. Bar 150 μm.

Figure 8 shows rat 3, 85 weeks on OTA consumption. Notably, there is faint TUNEL staining in nephron epithelial fragments of the kidney cortex, A being more peripheral, reflecting a situation like that in rat 1 (above), on a much lower OTA dosage here.

### 2.3. Fischer Rat—Second Year OTA Only

**Figure 9:**
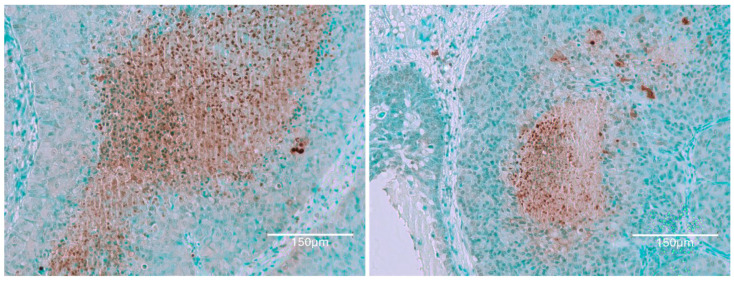
This tissue sample is only an extensive renal tumour, with patches with dark TUNEL-brown staining in nuclei. Inclusion here shows that OTA can generate tumour even when given later in life. Bars 150 μm.

Figure 9 shows OTA exposure (5 ppm in diet) only from one year of age, lasting then for only 35 weeks [12]. Inclusion here shows that OTA can generate renal tumour even when given late in life.

**Figure 10:**
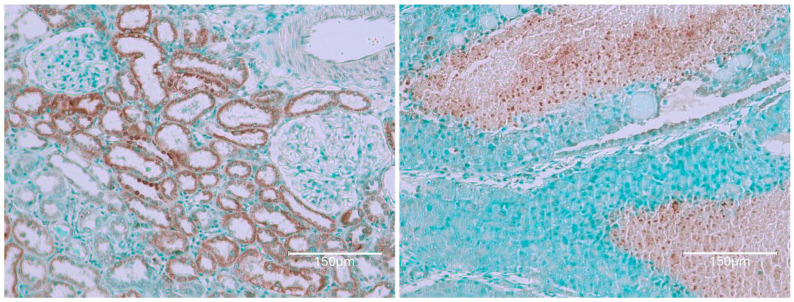
Typical TUNEL staining in renal cortex with notable nuclear emphasis in rows of nephron nuclei (**left**), and an example of renal tumour containing areas of mild TUNEL staining (**right**), included only for citation. Bars 150 μm.

**Figure 11:**
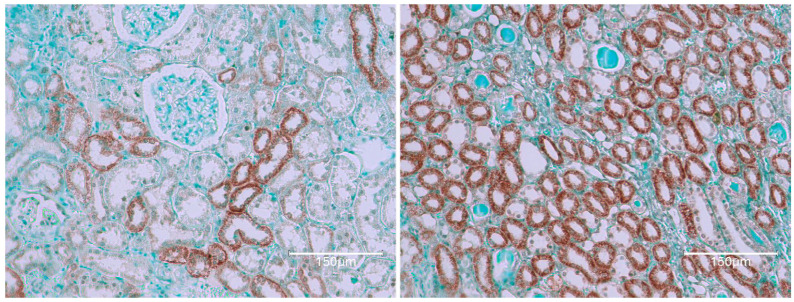
Fischer rat kidney cortex response to continuous OTA exposure only in 2nd year of life; familiar organised selective TUNEL staining of nephrons. Bars 150 μm.

Figure 10 shows a consumed 100 μg of OTA daily, only during the second year of life. Figure 11 demonstrates the kidney cortex response in Fischer rats following continuous OTA exposure, which is observed exclusively during the second year of life. The figure highlights the characteristic, organized, selective TUNEL staining of nephrons, indicating apoptotic activity within the cortical region.

### 2.4. Dark Agouti Rat—SINGLE RAT

**Figure 12:**
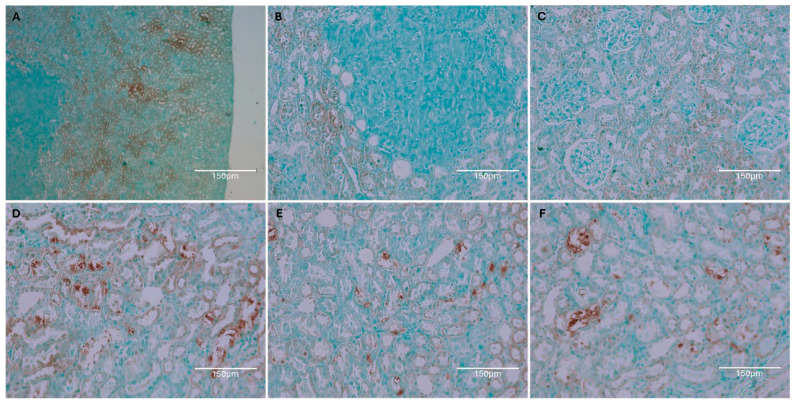
Dark Agouti single rat given OTA diet in first 6 months of life only. About 2 years later, a representative illustration of TUNEL staining in tumourous kidney; lower-power magnification shows contrast between renal cortex and small tumour (**A**); higher-power contrasts emphasise negative TUNEL evidence in tumour (**B**); renal cortex detail (**C**–**F**) illustrating very faint outer cortex staining in C and occasional intensities in inner cortex (**D**–**F**) but nuclear destination unclear. Bars 150 μm.

OTA exposure at 5 ppm in the diet was administered solely during the first six months of adult life, followed by a normal diet for almost two years [13]. Eventually, tumors developed in both kidneys, with the smaller one shown here [Figure 12].

The Figure 12 illustrates a rat strain with a longer natural life compared to modern white experimental rat varieties and with exposure to dietary ochratoxins at a rate similar to those in Section 2 above. Its revealed renal tumour allows some comparison with examples in Section 2 above. Abcam staining revealed a very microscopic renal tumour, completely free from TUNEL staining. This suggests that its origin was long after circulating renal OTA had become exhausted before embarking on the subsequent 2 years free from dietary OTA. Otherwise, only occasional fragments of TUNEL staining persisted among nephron fragments and appear disorganised.

The above array of pictures was chosen only to illustrate any positive products of the application of the Abcam staining kit to a range of apparently typical rat renal tissue representatives. This was after the extensive continuous silent passage of OTA through their kidneys except perhaps near the end of life. Mostly, they were from the maximum dose magnification, just to emphasise observation on the nuclei in the multi-replicated selective filtration systems across the cortex of kidneys. In contrast, there was a clear illustration of the consistent absence of any TUNEL positivity in a rat kidney’s OTA tumour. The specialised Abcam histology kit ensured illustrations of clear contrasts. Its use for such general exploration across a range of available rat renal tissues has revealed interesting illustrations in response across the kidney’s design, normally to encourage animal health. It seems that an animal model for readily revealing rat urothelial tumour origin has yet to arise.

## 3. Discussion

Our current attempt at the interpretation of the TUNEL-influenced detection of microscopic nuclear cleavage is one of the first pointers to apoptosis. A parallel application to staining rat and human renal tissues, as first reflected from our former description in BEN tissues, should allow some critical comparison in the interpretation of results. The experimental context for interpreting the present TUNEL-based staining of rat renal examples of chronic OTA poisoning seems based on its male gender near-specificity.

Historic experimentation 40 years ago for human significance demonstrated renal tumorigenic male specificity exclusively in mice [14]. Shortly after, in rats, renal tumours were exceptionally rare for females in the first large comprehensive toxicology study [5] and then only for one dramatic case at the highest dose of OTA. Subsequently, just two small terminal adenomas were found microscopically among the 50 animals at that highest OTA dose. The immune profiles of these female cases were explored further, more recently [11,12]. Their gender tendency was associated with some novel rodent-gender-linked vascular features, also relatively recently illustrated for female rats [15,16]. Their relevance for the present topic is slightly difficult because BEN has been evident rather more in women, and the reverse occurs very much more for OTA in experimental rats. However, the present illustration of OTA in rats has already been briefly shown for direct comparison with some specially illustrated Romanian renal examples of BEN [17]. It contained a brief rat example to forecast the present study, simply to further extend connectivity with the same examples of the rat histopathology.

For rats, this study just focuses on the display of TUNEL revelations in kidneys, made available since prominent in the renal cortex. This indicates the complexity and some effect of OTA’s vascular journey, travelling through kidney to accompany important biochemicals and allowing that vascular organ’s high selectivity function to maintain the demands of all of a rat’s active life. Thus, a two-fold rat gender difference concerning a maximum stable OTA circulation in blood is illustrated from its standard experimental voluntary dietary access [18]. This took about a month of constant consumption to stabilise. Thereafter, female rats tended to maintain the toxin quite efficiently during circulation by linking it loosely to blood proteins. Male rats link similarly but must compromise because of their gender’s additional medium-sized vascular peptides, with roles also of transporting small molecules with key sexual attractiveness functions. Appreciating that has been a third millennium discovery [19]. Then, it has been studied further for its current application concerning OTA toxicity [20].

Exploring this topic in London about 20 years ago [21], also described in this journal, had involved purchasing a primary antibody for OTA from R-Biopharm Rhone Ltd., Glasgow, UK. This formed a timely basis for imagining a male rat’s small vascular peptides transporting sexuality factors and to continue imagining that key role for OTA within nephrons. In an additional OTA context, transport of that mycotoxin into a descending renal nephron began the mycotoxin’s last journey. Within that nephron and progressing much more freely, the mycotoxin’s release from its small peptide transporter could then probably allow OTA’s structural tryptophan component to be salvaged. Such cleavage, and its salvage from within the nephron, would be an economic advantage since tryptophan cannot be synthesised by rats (or humans). The remaining OTA fragment is then ochratoxin alpha and travels directly out of the rat via the ureter. Thus, the original circulating molecule of OTA could possibly affect the kidney cortex while in brief fluid transit to the bladder. However, we are not aware that OT-alpha can cause urothelial tumours in rats; such does not seem to have been reported. Sections of OTA-created tumours within the rat kidney cortex are displayed here and had been made readily during the silent exposure to that mycotoxin for at least half a year. Such may still be a general principle for managing the understanding of OTA circulation and consequential toxicity. Variation between rat strains and genders can of course be expected. Conveniently, the dimensions of the chosen sections come from the organ’s shape and provide maximum illustration of its normal and tumorous components for standard study.

Previous experiments [22], designed to reveal aspects of delivery of circulating OTA within rat kidneys, showed maximum plasma concentration reached within about 3 h of acute gentle gavage but persisted for 4 days before decline. During long-term daily administration in feed, plasma values stabilised proportional to dose. For Fischer males, there followed an 8–10 day circulatory half-life when OTA delivery ceased. In mature Fischer males, plasma OTA accumulated during the month after starting the daily intake of 100 μg. In the smaller Dark Agouti males, lower steady-state plasma OTA values occurred, and the plasma half-life of OTA was only 2–3 days. Hybrid males accumulated OTA, but their females accumulated twice as much after 5 months of exposure. Any gender variation in humans could be interesting.

Concerning the detail of TUNEL staining here of rat kidney after many experimental months of quiet dietary OTA exposure, a similar illustration had been found in a Turkish publication a decade ago [23]. The helpful view in Figure 3 reveals caspase-3 reactions in male rat kidney histology several weeks after a period of daily dosing of OTA. Figure 4 there closely matches Figure 1 here. This raises an important comparison of the Abcam TUNEL kit used here with the staining used in the Turkey experiment. Both revealed similar renal tissue responses to dietary OTA. That histology was to demonstrate any caspase-3 reactions using a streptavidin-biotin peroxidase technique (Neomarker-California caspase 3 (CPP32) Ab-4 1:100 dilution). Sections had been developed using 3.3 diaminobenzidine as the chromogen.

Further comparative experimentation in Turkey [22], on an OTA diet at 5 ppm administered separately to both male and female rats, commenced at 16 weeks of age. After 6 weeks, the testes of a group of males were removed, but the OTA diet continued to all for 18 more weeks. All rats were then a novel source for organ and plasma analysis. Notably, female plasmas had about 12.5 μg of OTA/mL, but males had only half of that concentration. Extending that comparison with mice, their males also have vascular peptides of the 20 K size for expressing their sexuality.

In contrast, male rats and mice have several renal peptides of the 20 K size, to which sexuality factors bind. Some other small molecules such as OTA also link loosely, and complexes can be readily transferred into the nephrons in the rats’ kidney outer cortex. Once there, they can no longer logically circulate before gradual excretion. Notably, that immediate point of transfer remains unstained by the TUNEL kit.

Another contrasting publication in *IJMS*, concerning aristolochic acid as the local expected cause of BEN, chose young, 5-week-old, male rats to be injected with a dose intraperitoneally at 20 mg/kg of body weight. Its metabolic fate was explored after 2 further days of fasting [24]. Of course, the background of the Belgian women’s AA problem had been for adults, and BEN has naturally also been a little more abundant in females. Thus, an OTA-like accumulated dose of AA would need quite a lot of it for women. Where might tumour arise from AA within the abdomen of male rats? Forming DNA adducts is claimed to model BEN, even though by injected delivery into the abdomen and avoiding natural metabolic initial access to the liver, there might be more direct experimental access towards urothelial access to cause urological cancer. As a model, that single day’s dose was about 10-fold greater than that of the OTA illustrated here but was then received daily during the year to produce renal cancer silently. The literature quoting long-term gentle lifetime oral exposure to aristolochic acid for male and female rats, as for OTA, seems unfortunately not available. This could be because massive exploratory two-year studies would be necessary to match that for OTA in the USA 40 years ago [5]. That seems not to have occurred since the Belgian human epidemic highlighted the toxicity of aristolochic acid in the 1990s.

UK’s linkage with BEN commenced up to about 50 years ago, partly via experimental focus on mycotoxins [25] and also the revelation in Denmark of its annual seasonal risk to the major pig industry from home-grown feed that was poorly stored from harvest. A personal welcome for P.M. to Yugoslavia in November 1989 included a visit to the hyperendemic rural BEN village of Kaniza and to the nearby Slavonski Brod hospital that provided BEN’s renal-gavage medical help. Home-grown agricultural products from two Kaniza houses with a history of BEN provided examples for fungal analysis [26], revealing several *Penicillium* spp., of which *P. aurantiogriseum* producing OTA was most abundant. Rat renal toxicity of these fungi was also reported for their dietary exposure, and further experimental focus on potential nephrotoxicity in BEN became shifted to OTA.

In contrast, the serious unique women’s health epidemic in Belgium in the 1990s was treated with an Asian herbal medication to control extra weight. Its accidental content of aristolochic acid caused severe illness and death, but the measure of its contamination remained obscure. However, the suspicion of BEN being linked with aristolochic acid was caused. Subsequently, an experiment gave rabbits a daily intraperitoneal injection of aristolochic acid 5 days a week for up to 3 years and produced histopathology that claimed to be close to that of BEN [2]. This contrasts to that of stress-free lifetime dietary experimentation with OTA for rats, illustrated in the present study and has caused no urothelial tumours.

More recently, rat experiments [27] focused on the single peritoneal injection of aristolochic acids, followed by starvation for 2 days before killing. Their metabolic fate was based on preliminary experimentation [28] to establish the animal methodology, generous aristolochic dosage, initial digestive avoidance, direct peritoneal insertion, and 2 days of post-dosage starvation.

Early this millennium, an American lady used an herbal health remedy containing an *Aristolochia* component [9]. The duration of that exposure is unstated but, 3 years after ceasing it, terminal bilateral nephrectomy revealed renal adducts attributed to the aristolochic acid renal toxin, detected across renal tissues. Such histopathology was attributed to excessive exposure to this plant toxin, to which had been already attributed the severe responses in the Belgian women’s epidemic of the 1990s. Further studies in New York involved urothelial tissues of Romanian cases of BEN, which may have had features in common with those who used both for defining their anatomy and immunity profiles [8] and our recent human application of this Abcam apoptosis protocol [10]. Extensive analysis of these BEN tumours had revealed genetic conversions based on A-T → T-A transversions, detected in the late stage of BEN. This implied having made scientific discovery, implying human access to its genetic influence. Such changes were probably relatively late in the many years of the silent process of BEN atrophy, in which the revised features of genetic change had arisen as a secondary feature. Thus, it is thought to be difficult to claim such, as being a feature of the start of the renal decline of each BEN disease case amongst local groups of Romanian residents. Renal tumours seemed to be a secondary aspect of BEN, when it did not start the renal tissue decline.

Concurrently, research across several Balkan localities was conducted several years ago to be aware of local European context and express the findings informally [28] based on wide academic nephropathy experience.

Rat experimentation has been made on the belief that the natural botanical aristolochic acid complex in its two structural forms could illustrate its pair of natural causes of BEN [29]. Young male rats (170 g) were given ~7 mg of the natural aristolochic acids in 0.5 mL of corn oil by intraperitoneal injection. No food was then given for 2 days, while urine was collected for analysis. Notably, therefore, aristolochic acids could be available like OTA for male rodent urinary nephron transportation. Another experiment uses two forms [30].

A first journal’s illustrated claim to apoptosis in male rat kidney in response to chronic exposure to OTA [5] displayed the illustrated fluorescence in the cortico-medullary tissue, but subsequent online access to Nature is unfortunately without colour. A further technique to illustrate apoptosis initially used Guy’s Hospital London facilities and continued at Imperial College with methodology described since 1994 [31]. Its recent illustration [32,33] describes apoptosis in rat kidney tissue of a *Penicillium polonicum* containing OTA, a fungus resembling that isolated 30 years before from the Kaniza BEN village in Croatia.

Our exploration of historic studies on the cause of BEN and microbial factors offering logical involvement in its causes has revealed apoptotic findings and the challenge of elderly human renal tissues. The historic character of BEN cases, seen over about 70 years, was cited often in several politically isolated Balkan countries. More recently, their unique natural incidence seems to have declined, but scientific explanation for their origin has expanded within Europe, with various scientific explorations at molecular level. Publications frequently claim firm progress, but there remains a wide bridge between archived human tissue responses and the molecular explanation of their origin. Aristolochic acid is currently the popular agent for explaining BEN, but how it is actively claimed in Balkan countries is often scientifically hypothetical.

In 1991, a historical film was made in England for TV display and is still available online [34] to illustrate both the background and occasional mistaken causation. Its parallel addition of English translation of some human statements is sometimes curiously displayed in reverse on the internet, but the film uniquely conveys its 1950-90 local Balkan history.

There is yet no explanation of how toxic interaction within the human renal cortex can initiate the quite common urothelial renal tumour, as a typical feature of BEN. The current rat illustration of any TUNEL-recognised early step of tumour genesis by a hypothetical carcinogen of plant or microbial origin is a challenging topic. Meanwhile, there still seems to be no correction in the literature of the ~8-fold exaggeration of a commonly predicted contamination of human diet by aristolochic acid in BEN villages, mentioned among other serious critiques [33].

The wide range of common fungi producing secondary metabolites of unexplored toxicity was used again to explore renal apoptosis in *Penicillium polonicum* of Central European origin [34,35]. The application of a TUNEL assay revealed extensive histopathology in rats, illustrated in colour, confirming visual revelation of responses, complementary to those shown here by the Abcam system.

## 4. Materials and Methods

The TUNEL assay kit (ab206386; Abcam, Cambridge, MA, USA) was used according to the manufacturer’s instructions and had been used concurrently with our BEN tissues [10]. The key steps in this protocol, along with slight modifications for the ease of following, are given below.

### 4.1. Tissue Section Preparation and Rehydration

Paraffin-embedded kidney tissue section slides were immersed in xylene for two cycles of 5 min each at room temperature (RT), with frequent changes in xylene. Slides were transferred to 100% ethanol for two cycles of 5 min each at RT, followed by immersion in 90%, 80%, and 70% ethanol for 3 min each at RT. Slides were briefly rinsed with 1× Tris-buffered saline (TBS) for 5 min, and excess liquid was carefully dried around each specimen using Kimwipe laboratory wipes (Kimberly-Clark, Roswell, GA, USA) while ensuring that the specimen was circled with a hydrophobic slide marker to contain reagent volumes and also to avoid drying of specimen.

### 4.2. Permeabilization of Specimen

To permeabilize each specimen, Proteinase K was diluted 1/100 in deionized water (dH_2_O) (1 μL of Proteinase K + 99 μL of dH_2_O per specimen). Each was covered with 100 μL of the diluted Proteinase K solution and incubated at RT for 20 min. Following incubation, slides were rinsed with 1× TBS for 5 min.

### 4.3. Quenching: Inactivation of Endogenous Peroxidases

Endogenous peroxidases were quenched by diluting 30% hydrogen peroxide (H_2_O_2_) 1/10 in methanol (10 μL 30% H_2_O_2_ + 90 μL of methanol per specimen). Specimens were covered with 100 µL of 3% H_2_O_2_ and incubated at RT for 5 min. Subsequently, slides were rinsed with 1X TBS for 5 min. Specimens were covered with 100 μL of Terminal deoxynucleotidyl transferase (TdT) Equilibration Buffer and incubated at RT for 30 min.

#### 4.3.1. Labelling Reaction

Labelling enzyme was mixed with TdT Labelling Reaction Mix in a 1:39 ratio and applied to each specimen (40 μL per specimen). Slides were covered with a coverslip to ensure even distribution of the reaction mixture and prevent evaporation during incubation. Specimens were incubated in a humidified chamber at RT for 1.5 h.

#### 4.3.2. Termination of Labelling Reaction

Stop Buffer was warmed to 37 °C for 5 min. and then applied to each specimen (100 μL per specimen). Slides were incubated at RT for 5 min, rinsed with 1X TBS for 5 min, and excess liquid was carefully dried around the specimen. Specimens were then covered with 100 μL of Blocking Buffer and incubated at RT for 10 min.

#### 4.3.3. Detection

Conjugate was diluted 1/25 in Blocking Buffer (4 μL of 25X Conjugate + 96 μL of Blocking Buffer per specimen) and applied to each specimen. Slides were incubated in a humidified chamber at RT for 30 min, rinsed with 1X TBS for 5 min, and excess liquid was carefully dried around each specimen.

#### 4.3.4. Development

DAB solution was prepared by diluting DAB Solution 1 in DAB Solution 2 (1/30 dilution). Specimens were covered with 100 μL of diluted DAB solution and incubated at RT for 15 min. Slides were then gently rinsed with deionized water.

#### 4.3.5. Counterstain and Storage

Specimens were then immediately covered with 100 μL of Methyl Green Counterstain solution and incubated at RT for 1–3 min. Excess counterstain was removed by pressing an edge of the slide against an absorbent towel, and slides were dipped 2–4 times in 100% ethanol, and, then, slides were dipped 2–4 times in 100% xylene. Excess xylene was wiped off, and a glass coverslip was mounted using organic mounting media over the specimen.

An apoptosis end point, indicative of positive staining, was represented by a dark brown (DAB) signal. Lighter shades of brown and/or shades of blue-green to green-brown indicated a nonreactive/negative cell. After performing the assay, slides were evaluated under fluorescent microscope (M5000, Thermo Fisher Scientific; Pittsburgh, PA, USA) with brightfield capability. I imaged slides at 4×, and 20× magnifications. In addition, a Zeiss Axio Scan Z.1 Slide Scanner (Carl Zeiss Microscopy GmbH, Oberkochen, Germany) was utilised to digitise slides. This scanner generated slide scanning data over an extended period. The enclosed system scanner has 25 slide holder trays, and each tray has 4 slide slots so 100 slides can be uploaded to scan. The average size of images generated through Zeiss Axio Scan Z.1 Slide Scanner were ~1 GB and therefore were converted to smaller file formats like JPG for further analysis.

Application of this Abcam kit staining methodology, cited in over 150 publications, has nuclear apoptosis staining illustrated on its website. The present imaging at 4× and 20× magnification has provided illustrations for more representative description. The present staining could now indirectly link to the immune profiles of urothelial tumours in Slovakia [15] where BEN apparently has not occurred. The above methodology description is complementary to that recently published by us concerning the corresponding studies on BEN cases [10].

## Data Availability

The original contributions presented in this study are included in the article. Further inquiries can be directed to the corresponding author.
